# Influence of Maternal Carbohydrate Source (Concentrate-Based vs. Forage-Based) on Growth Performance, Carcass Characteristics, and Meat Quality of Progeny

**DOI:** 10.3390/foods10092056

**Published:** 2021-08-31

**Authors:** Erin R. Gubbels, Janna J. Block, Robin R. Salverson, Adele A. Harty, Warren C. Rusche, Cody L. Wright, Kristi M. Cammack, Zachary K. Smith, J. Kyle Grubbs, Keith R. Underwood, Jerrad F. Legako, Kenneth C. Olson, Amanda D. Blair

**Affiliations:** 1 Department of Animal Science, South Dakota State University, Brookings, SD 57007, USA; robin.salverson@sdstate.edu (R.R.S.); adele.harty@sdstate.edu (A.A.H.); warren.rusche@sdstate.edu (W.C.R.); cody.wright@sdstate.edu (C.L.W.); kristi.cammack@sdstate.edu (K.M.C.); zachary.smith@sdstate.edu (Z.K.S.); judson.grubbs@sdstate.edu (J.K.G.); keith.underwood@sdstate.edu (K.R.U.); Kenneth.olson@sdstate.edu (K.C.O.); amanda.blair@sdstate.edu (A.D.B.); 2 Hettinger Research Extension Center, North Dakota State University, Hettinger, ND 58639, USA; janna.block@ndsu.edu; 3 Department of Animal and Food Sciences, Texas Tech University, Lubbock, TX 79409, USA; jerrad.legako@ttu.edu

**Keywords:** beef, carcass characteristics, carbohydrate source, fetal programming, maternal nutrition, meat quality

## Abstract

The objective of this research was to investigate the influence of maternal prepartum dietary carbohydrate source on growth performance, carcass characteristics, and meat quality of offspring. Angus-based cows were assigned to either a concentrate-based diet or forage-based diet during mid- and late-gestation. A subset of calves was selected for evaluation of progeny performance. Dry matter intake (DMI), body weight (BW), average daily gain (ADG), gain to feed (G:F), and ultrasound measurements (muscle depth, back fat thickness, and intramuscular fat) were assessed during the feeding period. Carcass measurements were recorded, and striploins were collected for Warner-Bratzler shear force (WBSF), trained sensory panel, crude fat determination and fatty acid profile. Maternal dietary treatment did not influence (*p* > 0.05) offspring BW, DMI, ultrasound measurements, percent moisture, crude fat, WBSF, or consumer sensory responses. The forage treatment tended to have decreased (*p* = 0.06) 12th rib backfat compared to the concentrate treatment and tended to have lower (*p* = 0.08) yield grades. The concentrate treatment had increased (*p* < 0.05) a* and b* values compared to the forage treatment. These data suggest variation in maternal diets applied in this study during mid- and late-gestation has limited influence on progeny performance.

## 1. Introduction

Recent advances in fetal programming research indicate that altering maternal nutrition during the fetal stage can result in altered offspring productivity measures, including growth, feed intake, feed efficiency, muscle development, and meat quality [1]. Within the first two months of conception in the ruminant, development of adipocytes (fat tissue) and fibroblasts (connective tissue) occur along with development of skeletal muscle cells, all of which are primarily derived from mesenchymal stem cells [2].

Development of intramuscular fat, or marbling, is of great economic importance to the U.S. beef industry. Adipogenesis is initiated around the fourth month of gestation, partially overlapping with the second wave of myogenesis [2]. This stage of development represents an opportunity for maternal nutrition to positively or negatively affect stem cell differentiation [2]. Since the number of mesenchymal stem cells decrease as cattle mature, strategies to increase marbling during early life could be advantageous to improving meat quality. After 250 days of age, marbling is primarily enhanced only through the growth of preexisting adipocytes and nutritional influences have little impact on adipocyte development [3]. Further, different regulatory processes control fatty acid synthesis in intramuscular and subcutaneous adipose tissue, indicating that it may be possible to increase marbling without proportional increases in backfat that could negatively impact yield grades [4]. Thus, the fetal stage may be of key importance to programming carcass quality.

Volatile fatty acids (VFA) are the main products of the digestion of feed by bacteria in the rumen, provide a majority of the energy required by ruminants, and serve as substrates for synthesis of glucose and fat [5,6]. Major VFA produced by rumen microorganisms include acetate, propionate, and butyrate [6]. Various dietary carbohydrates ferment in the rumen to yield differing proportions of specific short- and long-chain fatty acids. Forage-based diets result in VFA composition of approximately 65 to 70% acetate, 15 to 25% propionate, and 5 to 10% butyrate in cattle [7]. Grain-based diets high in readily fermentable carbohydrate (starch) reduce acetate by 10 to 15% and increase propionate by 20 to 25% [7]. Propionate is the only VFA that contributes directly to the net synthesis of glucose, which is a major energy substrate utilized by uterine and placental tissues for fetal growth [5]. Although ruminal VFA production in gestating cows was not determined in the present study, it is plausible that diets based on nonstructural carbohydrates (starch), found in concentrate-based diets, rather than structural carbohydrates (fiber), found in forage-based diets, could influence fetal development and subsequent composition of the developing calf by way of altered VFA production profiles.

From a production perspective, management decisions made in response to drought, availability of feedstuffs, or cost of feedstuffs can alter the gestational environment, potentially leading to changes in fetal development. Previous literature has shown that providing first-calf heifers and mature cows with a high-energy diet 100 d prepartum increased body weight before parturition and calf birth weight [8]. In the study by Corah et al. [8] subsequent weaning weight was heavier for calves from cows consuming the high-energy diet. However, it has been reported feeding corn to dams in late pregnancy resulted in offspring with reduced marbling scores, a tendency towards reduced intramuscular fat percentage, and more carcasses grading United States Department of Agriculture (USDA) Select compared to offspring from hay-fed cows [9]. Because fetal adipocyte differentiation and growth is initiated during mid-gestation, it is possible that different responses would be observed if maternal dietary treatments had been implemented earlier. Based on these results, there may be differences in nutrient utilization and performance of offspring from cows fed forage or concentrate-based diets. We hypothesized that variations in the proportion of volatile fatty acids produced in the rumen of the gestating cow caused by differing dietary carbohydrate sources during mid- and late- gestation would differentially influence fetal development and offspring composition, leading to alterations in performance and meat quality of offspring. The objective of this study was to investigate the effects of maternal prepartum dietary carbohydrate source (forage- vs. concentrate-based) during mid- and late-gestation on growth performance, carcass characteristics, and meat quality of offspring.

## 2. Materials and Methods

### 2.1. Cow Management

All animal care and experimental protocols were approved by the South Dakota State University (SDSU) Animal Care and Use Committee (approval number 18-081E). Mature, Angus-based, spring-calving cows (*n* = 131) from the SDSU Antelope Range and Livestock Research Station were evaluated for pregnancy in the fall of 2017 and assigned to dietary treatments based on cow age and body condition score (BCS). Groups were randomly assigned to a forage-based or concentrate-based dietary treatment and allotted to two pens based on treatment (Forage (*n* = 64) or Concentrate (*n* = 65)). The uterine environment created by differing VFA profiles within each cow was considered the experimental unit. Dietary composition of the treatment diets is provided in Table 1.

Feed intake was controlled so that cows in both treatments consumed equal amounts of protein and energy. Cows were provided the treatment diets beginning at approximately day 94 of gestation and continuing until approximately 30 days prior to calving. Both diets were formulated to maintain cow body condition. Body weight (BW) and BCS from the beginning (day 0) and end (day 98) of the treatment period were used to monitor the influence of dietary carbohydrate source on cow performance. Initial BW was recorded after a two-week diet adaptation period to account for differences in gut fill (cows were provided treatment diets that varied in digestibility and intake compared to the pre-treatment diet). Average initial BW of the cows was 598 ± 49.4 kg and 666 ± 52.4 kg for concentrate and forage treatments, respectively (likely due to differences in rumen fill), and average BCS was 5.2 ± 0.39 and 5.3 ± 0.31 for concentrate and forage treatments, respectively. At the completion of the treatment period the average BW of the cows was 639 ± 60.7 kg and 635 ± 57.4 kg, and average BCS was 5.4 ± 0.57 and 5.1 ± 0.38 for concentrate and forage treatments, respectively. At the end of the treatment period, cows were returned to native range pastures and managed as a common group through weaning.

### 2.2. Offspring Management

At approximately 60 days of age, all calves were vaccinated with a killed vaccine for clostridial diseases (Vision 7 Somnus with SPUR, Merck Animal Health, Madison, NJ, USA). At approximately 110 days of age, all calves were administered a modified-live vaccine for prevention of bovine rhinotracheitis (IBR), bovine viral diarrhea (BVD), bovine respiratory syncytial virus (BRSV) Types 1 and 2, and parainfluenza-3 (PI_3_), Haemophilus somnus, and *Mannheimia haemolytica* (Pyramid 5+ Presponse SQ, Boehringer Ingelheim Vetmedica, Inc., St. Joseph, MO, USA). At weaning, all calves were administered an anthelmintic (Dectomax Pour-On Solution, Zoetis, Parsippany, NJ, USA) and were provided boosters of the clostridial disease and respiratory disease vaccines. At this time, a subset of 96 calves (*n* = 24 heifers/treatment, *n* = 24 steers/treatment) closest to the mean weaning weight were shipped to the SDSU Cottonwood Field Station. Calves were fed a common receiving diet consisting of grass hay and dried distiller’s grains with solubles during an 83-d backgrounding period. On day 36 postweaning, calves were weighed and ultrasounded to determine backfat thickness (BF), muscle depth of the *longissimus dorsi*, and intramuscular fat (IMF) measured at the 12th and 13th rib.

At the conclusion of the backgrounding phase, all calves were transported approximately 526 km to Brookings, SD for the finishing phase of the study. Upon arrival, calves were vaccinated against clostridia perfringens type A (Clostridium Perfringens Type A Toxoid; Elanco, Greenfield, IN, USA). The calves were finished in an Insentec monitoring system (Insentec, Marknesse, The Netherlands) to monitor individual feed intake (steers and heifers were fed separately in two pens) at the SDSU Cow-Calf Education and Research facility. Calves were stepped up to their finishing diets over 14-days; final diets are shown in Table 2. Diet ingredients were sampled weekly and monthly composites were used to determine the dry matter [10], crude protein [11], neutral detergent fiber [12], acid detergent fiber [13], ash [14], crude fat [15]. Tabular values for diet ingredients were used to calculate energy content of diets.

Cattle were weighed at 28-days intervals during the finishing period to monitor performance (hereafter referred to as Period 1, Period 2, etc.). Calves were administered an initial growth promoting implant on day 23 of the finishing period containing 100 mg trenbolone acetate (TBA) and 14 mg estradiol benzoate (EB) (Synovex-Choice, Zoetis Inc., Parsippany, NJ, USA). Cattle were re-implanted with 100 mg TBA and 14 mg EB (Synovex-Choice, Zoetis Inc., Parsippany, NJ, USA) and a second ultrasound was conducted on day 80 of the finishing period. Ultrasound measures collected during the backgrounding period and finishing period were compared to determine changes in composition. The second ultrasound was also used to predict harvest date. The harvest target was determined when the predicted BF was approximately 1.27 cm, resulting in three harvest dates at day 131, day 145, and day 180 of the finishing period. Cattle were weighed the morning of slaughter to determine final live BW and shipped 235 km to a commercial harvest facility.

### 2.3. Carcass Evaluation and Sample Collection

All cattle were tracked individually through the harvest process. Following carcass chilling (approximately 24 h), hot carcass weight (HCW), ribeye area (REA), 12th rib BF, USDA Yield Grade, marbling score, carcass maturity, and USDA Quality Grade were evaluated according to the United States Standards for Grades of Carcass Beef [16]. Objective color measurements (L*, a*, and b*) were also recorded at the exposed REA of each carcass using a handheld Minolta colorimeter (Model CR-310, Minolta Corp., Ramsey, NJ, USA; 50 mm diameter measuring space, D65 illuminant). A strip loin (IMPS #180) was collected from each carcass and transported to the SDSU Meat Science Laboratory, portioned into 2.54-cm steaks, and vacuum packaged. Four steaks were aged for either 3, 7, 14, or 21 days at 4 °C and then frozen at −10 °C for evaluation of Warner-Bratzler shear force (WBSF). Additional steaks were utilized to determine fatty acid profile using Fatty Acid Methyl Ether (FAME) synthesis, crude fat percentage using ether extraction, and consumer palatability of 14-d aged samples using a trained sensory panel.

### 2.4. Warner-Bratzler Shear Force

Steaks designated for WBSF determination were thawed for 24 h at 4 °C then cooked on an electric clamshell grill (George Foreman, Model GRP1060B, Middleton, WI, USA) to an internal temperature of 71 °C. A thermometer (Model 35140, Cooper-Atkins Corporation, Middlefield, CT, USA) was used to record the peak internal temperature. Cooked steaks were cooled at 4 °C for 24 h before removing 6 cores (1.27 cm diameter) parallel to the muscle fiber orientation [17]. A single, peak shear force measurement was obtained for each core using a texture analyzer (Shimadzu Scientific Instruments Inc., Lenexa, KS, USA, Model EZ-SX) with a Warner-Bratzler attachment. Measurements of the peak shear force value were averaged to obtain a single WBSF value per steak.

### 2.5. Ether Extract

At 3 d postmortem, the anterior face of each striploin was removed during fabrication and frozen at −20 °C and later used to determine percent crude fat using the ether extract method described by Mohrhauser et al. [18]. Steaks were thawed slightly and all exterior fat, epimysial connective tissue, and additional muscles were removed leaving the longissimus muscle for evaluation. Samples were minced, immersed in liquid nitrogen, and powdered for 15 s using a Waring commercial blender (Waring Products Division, Model 51BL32, Lancaster, PA, USA). Homogenized samples were weighed in duplicate 5-g samples into dried aluminum tins, covered with dried filter papers, and dried in an oven at 100 °C for 24 h. Dried samples were then placed into a desiccator and were reweighed after cooling. Samples were extracted using petroleum ether in a side-arm Soxhlet extractor (Thermo Fischer Scientific, Rockville, MD, USA) for 60 h followed by drying at room temperature and subsequent drying in an oven at 100 °C for 4 h. Dried extracted samples were placed into a desiccator for 1 h and were cooled and then reweighed. Crude fat was calculated by subtracting the pre-extraction weight from the post-extraction sample weight and expressed as a percentage of the pre-extraction sample weight.

### 2.6. Fatty Acid Composition

A sub-sample of 60 steaks (*n* = 30 steaks closest to the mean marbling score of each treatment) were selected to evaluate fatty acid profile using direct FAME synthesis. Steaks were thawed slightly and external fat, epimysial connective tissue, and additional muscles were trimmed from the longissimus muscle. Samples were minced, immersed in liquid nitrogen, and powdered for 15 s using a Waring commercial blender (Waring Products Division, Model 51BL32, Landcaster, PA, USA). Duplicate 1 g samples were weighed and processed to generate FAMEs according to procedures of O’Fallon et al. [19]. Fatty acids were identified through comparison with retention times of an authentic fatty acid standard mixture (GLC-463, Nu-Check Prep Inc., Elysian, MN, USA). Quantities were computed as mg/g of raw wet tissue through an internal standard calibration method where C13:0 served as the internal standard. Final contents were then summed and %, g/100 g total fatty acids was produced after summing all fatty acids.

### 2.7. Trained Sensory Panel

The human sensory panel utilized in this study was approved by the Institutional Review Board of South Dakota State University (IRB-1911019-EXM). Eight sensory panelists were trained to evaluate meat quality attributes of strip loin steaks according to the American Meat Science Association training guidelines appropriate for the study [17]. Panelists were 18 years or older, had no food allergies or sensitivities, and had consumed any type of meat products at least once a year. Strip loin samples were evaluated for juiciness (1 = extremely dry; 18 = extremely juicy), tenderness (1 = extremely tough; 18 = extremely tender), and beef flavor (1 = extremely bland; 18 = extremely intense) on an anchored unmarked line scale. Steaks were cooked on an electric clamshell grill (George Foreman, Model GRP1060B, Middleton, WI) to an internal temperature of 71 °C. After cooking, steaks were rested for five minutes and then cut into 2.5 × 1 × 1-cm samples. Two cubes were placed into a prelabeled plastic cup, covered with a plastic lid in order to retain heat and moisture, and held in a warming oven (Metro HM2000, Wilkes-Barre, PA, USA) at 60 °C until served. Evaluations were performed according to American Meat Science Association guidelines [17]. Ten samples were evaluated in each session, one session per d, for a total of 10 sessions. Samples evaluations were alternated by treatment to reduce first and last order bias. Samples were served to panelists in a randomized fashion, in private booths, under red lights to limit observation of visual differences.

### 2.8. Statistical Analyses

Response variables were analyzed using generalized linear mixed model procedures (SAS GLIMMIX, SAS Inst. Inc., Cary, NC, USA) in a completely randomized design. The intrauterine environment was considered the experimental unit for ultrasound measurements, carcass characteristics, and meat quality data and was designated as a random effect. Treatment, sex, and their interaction were included in the model as fixed effects. For carcass characteristics and meat quality data, harvest date was included in the model as a fixed effect to absorb variation due to this effect (data not shown). For WBSF, aging period was added to the model as a repeated measure and peak cooking temperature was included as a covariate. Separation of least squares means was conducted using protected LSD. Treatment by sex interactions were evaluated and discussed if significant.

## 3. Results

### 3.1. Growth Performance

Animal performance and growth data are reported in Table 3. Maternal dietary treatment did not influence (*p* > 0.05) offspring BW, or DMI. In Period 1 (day 0–23) of the finishing phase, offspring from dams fed a forage-based diet tended (*p* = 0.079) to have an improved ADG compared to the offspring from dams fed a concentrate-based diet.

**Figure 1:**
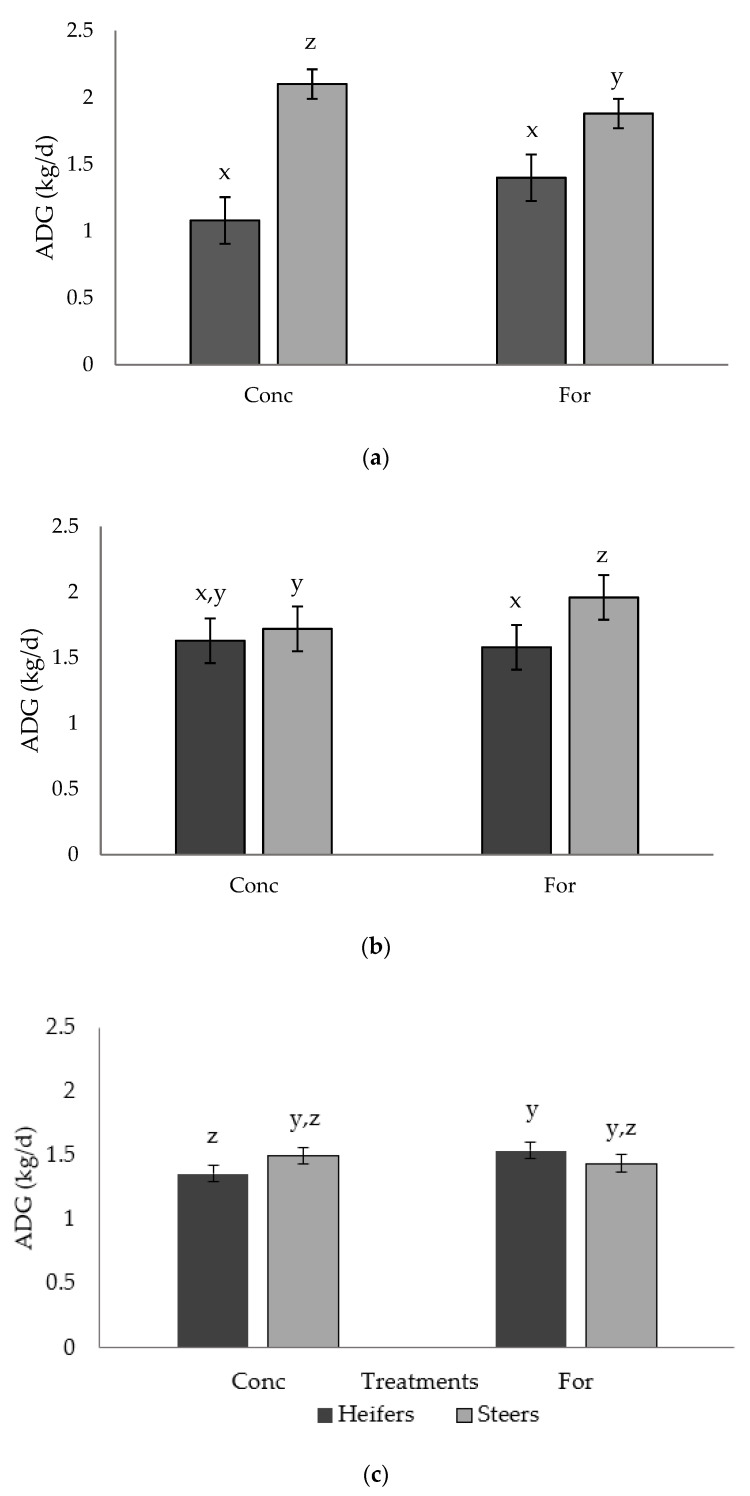
Treatment by sex interaction for ADG (kg/d) of progeny in: (**a**) Period 2 (*p* = 0.054), (**b**) Period 3 (*p* = 0.071), and (**c**) Period 5 (*p* = 0.067) from dams fed a concentrate-based (Conc) or forage-based (For) diet during mid- and late-gestation. Diets formulated based on NRC (2000) requirements for dams fed either a concentrate or forage diet during mid- and late-gestation. x, y, z LSmeans lacking a common superscript differ (*p* ≤ 0.05).

A tendency (*p* = 0.054) for a treatment × sex interaction was detected for ADG in Period 2 (Figure 1a). Steers from the concentrate treatment had greater (*p* < 0.04) ADG compared with steers from the forage treatment, while ADG of heifers did not differ (*p* > 0.05) between treatments. A tendency (*p* = 0.071) for a treatment × sex interaction was also detected for ADG in Period 3 (Figure 1b). 

Steers from the forage treatment had greater (*p* < 0.04) ADG than steers from the concentrate treatment as well as the heifers from either treatment, which were similar (*p* > 0.05). A tendency (*p* = 0.067) for a treatment × sex interaction was observed for ADG in Period 5 (Figure 1c). Steers from both treatments had similar (*p* > 0.05) ADG, and had similar (*p* > 0.05) ADG compared to both forage and concentrate heifers; however, forage heifers tended to have improved (*p* = 0.06) ADG compared to concentrate heifers.

**Figure 2:**
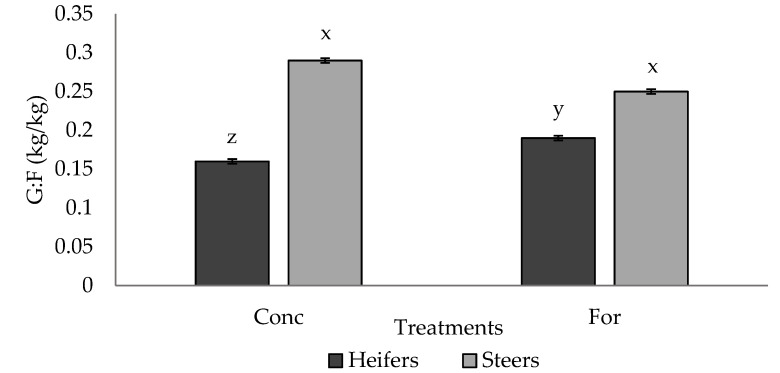
Treatment by sex interaction (*p* = 0.065) for G:F (kg/kg) of progeny in Period 2 from dams fed a concentrate-based (Conc) or forage-based (For) diet during mid- and/or late-gestation ^1^. ^1^ Diets formulated based on NRC (2000) requirements for dams fed either a concentrate or forage diet during mid- and late-gestation. ^x, y, z^ LSmeans lacking a common superscript differ (*p* ≤ 0.05).

No differences (*p* > 0.05) in G:F were observed between treatment groups; however, a tendency (*p* = 0.065) for a treatment × sex interaction was detected for G:F in Period 2 (Figure 2). Steers from both treatments had similar (*p* > 0.05) G:F, and had improved (*p* < 0.05) G:F compared to heifers from both treatments, however the forage heifers tended to have improved (*p* = 0.09) G:F compared to the concentrate heifers.

As expected, steers had greater (*p* < 0.05) BW compared to heifers at all time periods and had an increased (*p* < 0.05) ADG from day 37–83. Steers also had increased (*p* < 0.05) ADG in Period 2 (day 23–51) and Period 3 (day 51–78) compared to heifers. However, heifers had an increased (*p* < 0.05) ADG in Period 4 day 78–106) and tended to have an increased (*p* = 0.051) ADG compared to steers in Period 1 (day 0–23). Heifers had greater (*p* < 0.05) DMI during Period 1, however, DMI did not differ (*p* > 0.05) between steers and heifers for the remainder of the finishing period. Steers had improved (*p* < 0.05) G:F during Period 2 (day 23–51) and 3 (day 51–78), while heifers had improved (*p* < 0.05) G:F during Period 4 (day 78–106). It is likely that differences in G:F were driven by differences in ADG rather than DMI.

### 3.2. Ultrasound Measurements

**Figure 3:**
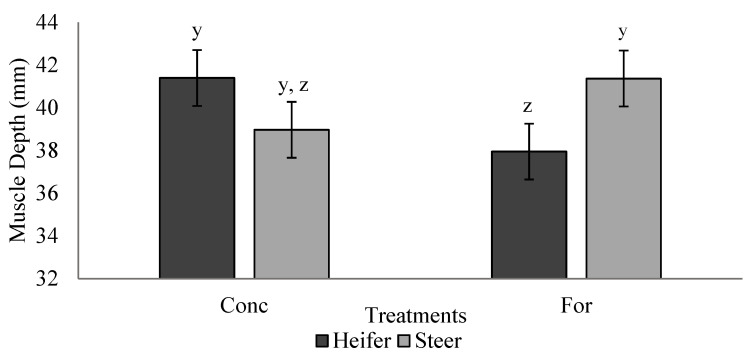
Treatment by sex interaction (*p* = 0.028) for muscle depth measured via ultrasound during backgrounding (d 36) of progeny from dams fed a concentrate-based (Conc) or forage-based (For) diet during mid- and/or late-gestation. Diets formulated based on NRC (2000) requirements for dams fed either a concentrate or forage diet during mid- and late-gestation. ^y,z^ LSmeans lacking a common superscript differ (*p* ≤ 0.05).

Ultrasound measurements are reported in Table 4. Maternal treatment did not influence (*p* > 0.05) offspring BF, IMF percentage or muscle depth during the finishing phase. A treatment × sex interaction (*p* = 0.028) was detected for muscle depth during the backgrounding phase (Figure 3). Heifers from the concentrate treatment tended to have increased (*p* = 0.07) muscle depth compared with heifers from the forage treatment, while muscle depth of steers did not differ (*p* > 0.05) between treatments. Heifers had increased (*p* < 0.05) BF compared to steers at the initial ultrasound during the backgrounding phase.

### 3.3. Carcass Characteristics

Carcass measurements are reported in Table 5. Maternal treatment did not influence (*p* > 0.05) offspring HCW, REA, marbling score, L* values, or the proportion of carcasses in each USDA Quality and Yield Grade category. Offspring from the forage treatment tended to have decreased (*p* = 0.060) 12th rib fat thickness and tended to have lower (*p* = 0.084) USDA Yield Grades compared to offspring from the concentrate treatment. Offspring from the concentrate treatment had increased (*p* < 0.05) a* and b* values compared to the forage treatment. As expected, steers had heavier (*p* < 0.05) HCW and larger (*p* < 0.05) REA than heifers. Heifers had increased (*p* < 0.05) BF and marbling scores, as well as increased (*p* < 0.05) a* and b* values and tended (*p* = 0.070) to have higher USDA Yield Grades.

### 3.4. Meat Quality Characteristics

Meat quality characteristics are reported in Table 6. Maternal treatment did not influence (*p* > 0.05) crude fat percentage, moisture content, WBSF, or sensory characteristics of steaks from offspring. Heifers had decreased (*p* < 0.05) moisture and increased crude fat content compared to steers and tended (*p* = 0.068) to have improved WBSF values compared to steers. No differences (*p* > 0.05) were detected between steers and heifers for sensory characteristics of steaks. As expected, WBSF improved (*p* < 0.05) with each aging period (4.75 ± 0.152 kg, 3.79 ± 0.112 kg, 2.98 ± 0.088 kg, and 2.65 ± 0.064 kg for steaks aged 3, 7, 14, and 21 days, respectively).

### 3.5. Fatty Acid Composition

Fatty acid composition data is reported in Table 7 and Table 8. The concentration (mg/g wet raw tissue; Table 7) of arachidonic (C20:4n6), nervonic (C20:1n9), and docosapentaenoic (C22:5n3) acids were increased (*p* < 0.05) in samples from the concentrate treatment; however, treatment did not influence (*p* > 0.05) concentration of other fatty acids. The concentration of capric (C10:0), myristic (C14:0), myristoleic (C14:1n5), palmitoleic (C16:1n7), and heptadecenoic (C17:1) acids were increased (*p* < 0.05) in samples from heifers compared with steers. Sex did not influence (*p* > 0.05) concentration of other fatty acids.

When analyzed as a percentage of total fatty acids (%, g/100 g total fatty acids; Table 8), docosatrienoic (C22:3), nervonic (C24:1n9), and docosapentaenoic (C22:5n3) acids were increased (*p* < 0.05) in samples from the concentrate treatment compared with the forage treatment. Treatment did not influence (*p* < 0.05) the percentage of other fatty acids. The percentage of myristic (C14:0), palmitoleic (C16:1n7), and heptadecenoic (C17:1) acids were increased (*p* < 0.05) in samples from heifers compared with steers, but the percentage of stearic (C18:0) acid was increased (*p* < 0.05) in samples from steers. Sex did not influence (*p* > 0.05) the percentage of other fatty acids.

## 4. Discussion

The majority of fetal muscle and adipose tissue growth and development occurs during mid- and late-gestation [2]. Alterations to fetal development imposed by maternal stressors, such as maternal nutrient restriction have been shown to have long term impacts on offspring growth and performance [18,20,21]. Dietary carbohydrate sources (i.e., fiber vs. starch) alter molar proportions of ruminal VFA and overall production of VFA’s [4]. While this is well documented in the literature, ruminal VFA production was not determined in gestating cows used in the present study, presenting a limitation to the results presented herein. In the present study, drought conditions in 2017 resulted in limited forage availability at the SDSU Antelope Range and Livestock Research Station. Therefore, a management decision was made to transport a portion of the cow herd to a drylot from November 2017 through February 2018 to take advantage of lower cost feedstuffs and preserve range conditions. Based on feed prices in 2017, dams in the concentrate-based treatment were fed a diet that cost approximately $0.90/day and the forage-based treatment were fed a diet that cost approximately $1.07/day. Others have evaluated dietary energy source during late gestation [9], but to date literature concerning the effects of maternal dietary carbohydrate source (forage vs. concentrate) during mid- and late-gestation on offspring performance and meat quality traits is limited.

In agreement with the present study, Radunz et al. [9] reported that maternal energy source did not influence feedlot receiving BW, DMI, ADG, G:F, or final BW of offspring. Taylor et al. [22] also reported that maternal energy status (positive or negative energy status) during mid-gestation did not influence offspring BW, ADG, DMI, or G:F during the finishing phase. However, studies investigating maternal protein supplementation in late gestation have reported differences in offspring performance. Larson et al. [23] investigated the effects of winter grazing system and crude protein supplementation to dams during late gestation, and offspring weaning BW, BW at feedlot entry, reimplant BW, ADG, and DMI were all increased when dams were supplemented with protein during late gestation [23]. Summers et al. [24] compared dams provided a supplement with a high level of rumen undegradable protein (RUP) or a low level of RUP during late gestation to a non-supplemented control. Offspring from dams supplemented with a high level of RUP had increased BW at feedlot entry compared to progeny from non- supplemented dams. However, progeny from non-supplemented dams tended to have greater ADG and had greater DMI during the reimplant period as well as greater overall DMI [24]. Differences in growth performance between studies is likely due to differences in nutrients evaluated (energy vs. protein), timing of maternal dietary treatments during gestation, and varying degrees of restriction or supplementation. However, these studies indicate that growth performance of offspring is sensitive to changes in the maternal diet.

There was a tendency for muscle depth of heifers from the concentrate treatment to be greater (9% increase) compared to heifers from the forage treatment at the initial ultrasound during the backgrounding phase. As ultrasound measures were recorded shortly after the weaning event, this result may indicate that heifers from the forage treatment required longer to adjust to the backgrounding environment, hindering their muscle growth. However, no differences were detected at the finishing period ultrasound, which may be attributed to recovery of muscle growth via compensatory growth. Radunz et al. [9] provided dams either hay-based, corn-based, or dried corn distiller’s grains-based diets during late gestation and evaluated carcass measures of progeny via ultrasound at 24 to 72 h after birth and 84 d into the finishing phase. However, unlike the present study, no differences were reported in ultrasound measures of progeny carcass traits. Differences in diet composition, timing of dietary treatments during gestation, and timing of ultrasound evaluation may explain the differences between the findings of Radunz et al. [9] and the present study.

Backfat thickness of offspring from forage fed dams tended to be decreased by 7% and USDA Yield Grades also tended to be 7% lower than offspring from concentrate fed dams. While no direct comparisons with the present study are available in the literature, other research has demonstrated that offspring fat depots may be especially sensitive to alterations in the maternal diet. When fed to a common BF endpoint, Radunz et al. [9] reported that offspring from dams fed a fiber-based diet (hay) in late gestation had increased marbling scores and no carcasses that graded USDA Select compared to offspring from dams fed a starch-based diet (corn). Underwood et al. [21] reported that BF and adjusted 12th rib BF were increased in offspring from dams grazing improved pasture that provided more crude protein than offspring form dams grazed on native range during mid gestation. Wilson et al. [25] observed a tendency for progeny from dams provided a distiller’s grain supplement during late gestation to have decreased backfat thickness compared to progeny from dams that were not supplemented. Steers from dams fed supplemental protein during late gestation were reported to have increased marbling scores, as well as a greater proportion of carcasses grading USDA Choice or higher compared to steers from dams not supplemented protein [23]. Mohrhauser et al. [18] reported a tendency for decreased BF and lower USDA Yield Grades, with no influence on marbling score, in offspring from dams in a negative maternal energy status during mid-gestation compared to offspring from dams in a positive maternal energy status. Summers et al. [24] also observed decreased 12th rib fat thickness with no differences in marbling score in progeny from dams that were supplemented a diet with low RUP in late gestation compared to progeny from dams not supplemented with RUP.

Heifers had increased BF (14%) and USDA Yield Grade (7%) compared to steers but decreased HCW (9%) and REA (8%). Mohrhauser et al. [18] also reported steers to have heavier HCW, reduced marbling scores, and larger ribeye areas. However, in contrast to the present study, steers were reported to have higher a* values and tended to have higher L* values compared to heifers [18]. In addition, the marbling score of heifers was greater (9%) compared to steers. This is consistent with other studies suggesting heifers have greater amounts of marbling when compared to steers and bulls [26].

Because there were no differences in marbling scores between treatment groups, the lack of difference in crude fat and moisture content is not unexpected. Other studies investigating alterations in maternal energy have evaluated WBSF and also reported no differences in this objective measure of tenderness [9,18]. However, studies investigating alterations in maternal protein levels reported steaks from offspring of dams with restricted protein intake during mid-gestation had increased WBSF values (less tender meat) compared to offspring of dams with adequate protein intake [20,21]. Other studies investigating the effects of maternal nutrition during gestation on sensory characteristics of steaks are lacking. Heifers had increased crude fat (25%) and decreased moisture content (2%) compared to steers, which is likely attributed to heifers having greater amounts of marbling.

There is limited information on the effects of maternal diet on the fatty acid composition of meat from offspring. Webb et al. [20] reported that arachidonic acid was sensitive to changes in maternal diet. Offspring of dams provided adequate protein during mid-gestation produced offspring with increased concentrations of arachidonic acid compared with protein restricted dams. A study by Chail et al. [27] evaluated the effects of finishing diet on fatty acid composition in the *gluteus medius* and *triceps brachii* and observed increased concentration of arachidonic acid when cattle were fed a grain-based diet as compared to a forage-based diet. In a recent review, Ponnampalam et al. [28] outlined that concentrate-based diets are common sources of omega-6 (n-6) polyunsaturated fatty acids compared to forage-based diets, which are common sources of omega-3 (n-3) polyunsaturated fatty acids. This is an important concern as current human dietary recommendations suggest a n6:n3 of 1–4:1. In the present study no differences were observed between treatment groups when n6:n3 fatty acid levels of progeny were evaluated. However, results from the present study suggest that maternal diet can influence fatty acid composition of steaks from progeny and warrants further investigation.

## 5. Conclusions

Results from this study suggest variation in maternal carbohydrate source during mid- and late-gestation has limited influence on progeny performance. Collectively, these data suggest a forage-based diet provided to cows during mid- and late-gestation differentially influences deposition of subcutaneous fat without compromising marbling score or tenderness. As dams in the present study were fed to meet nutrient requirements during mid- and late-gestation, mechanisms by which carbohydrate source in mid- to late-gestation can affect growth rate of progeny might be minimized when energy needs of the cow are met. Provided that nutrient requirements are met, it appears that utilizing alternative diets for the beef cow herd does not significantly influence progeny performance and beef product quality. Based on this study, cattle producers have flexibility to feed their gestating cows available carbohydrate sources during drought and/or variable growing conditions without concern for offspring performance or carcass traits.

## Figures and Tables

**Table 1:** Dietary components (dry matter basis) consumed by cows receiving a forage-based (For) or concentrate-based (Conc) diet during mid- and late-gestation.

Ingredient	Conc ^1^	For ^1^
Wheat Straw, %	24.1	71.9
Grass/Alfalfa Hay, %	0.0	21.8
Corn Silage, %	0.0	3.7
Suspension Supplement ^2^, %	4.6	2.6
Corn Grain, %	56.6	0.0
Modified Distiller’s Grain w/Solubles, %	13.3	0.0
Limestone, %	1.4	0.0
	Diet Composition	
Dry Matter Intake, kg	6.4	10.73
Dry Matter Intake, % BW	0.98	1.65
Roughage Intake, % BW	0.30	1.58
Crude Protein, % of DM	12.02	7.55
TDN, % of DM	73.18	50.88
NEm (Mcal/kg)	1.67	0.99
NEg (Mcal/kg)	1.05	0.46

^1^ Diets formulated based on NRC (2000) requirements. ^2^ Suspension supplement: 20% Crude Protein (≤20% Non-protein nitrogen), 3.55–4.55% Ca, 0.20% P, 0.30% Mg, 1% K, 528.63 ppm Mn, 12.65 ppm Co, 480 ppm Cu, 5.50 ppm Se, 1440 ppm Zn, 88184 IU/kg Vit. A, 24912 IU/kg Vit. D3, 165 IU/kg Vit. E, 400 g/ton monensin.

**Table 2:** Dietary components and nutrient composition of finishing diet ^1^ consumed by offspring of cows receiving a forage-based or concentrate-based diet during mid- and late-gestation.

Ingredient	% DM Basis
Grass Hay	10.77
Earlage	11.20
Dry Rolled Corn	53.85
Dried Distiller’s Grains w/Solubles ^2^	17.66
Suspension Supplement ^3^	6.51
	Nutrient Composition of Diet
DM %	72.00
CP %	14.61
ADF %	10.32
NDF %	20.74
Crude Fat %	3.74
Ash %	3.41
NEm (Mcal/kg)	2.05
NEg (Mcal/kg)	1.36

^1^ Diet formulated based on NRC (2000) requirements. ^2^ Dried distiller’s grains w/solubles fed to heifers included melengestrol acetate (MGA, Zoetis, Parsippany, NJ, USA) at a rate sufficient to provide 0.50 mg·hd^‒1^·d^‒1^; steers received dried distiller’s grains w/solubles without MGA. ^3^ Suspension supplement: 30.8% protein (26.6% non-protein nitrogen), 8% Ca, 0.2% P, 0.4% Mg, 7.1% K, 15.6 ppm Co, 337.6 ppm Cu, 33.8 ppm I, 723.8 ppm Mn, 3.2 ppm Se, 1107.8 ppm Zn, 9502 IU/kg Vit A, 2381 IU/kg Vit D3, 848 IU/kg Vit E, 512.3 g/ton monensin.

**Table 3:** Growth performance for progeny of dams fed a prepartum dietary carbohydrate source consisting of concentrate-based (Conc) or forage-based (For) diet during mid- and late-gestation.

	Treatment ^1^			Sex			*p*-Value ^2^		
	Conc	For	SEM ^3^	Heifers	Steers	SEM ^3^	Trmt	Sex	T × S
Backgrounding Phase									
Weaning BW, kg	281	277	3.7	272	286	3.7	0.475	0.009	0.951
Day 1–36									
Ending BW, kg	280	280	3.2	274	286	3.2	0.830	0.012	0.748
ADG ^4^, kg	−0.04	0.09	0.067	0.06	−0.01	0.067	0.166	0.495	0.735
Day 37–83									
BW, kg	321	321	3.4	309	333	3.4	0.994	<0.001	0.909
ADG ^4^, kg	0.86	0.84	0.042	0.74	0.96	0.042	0.738	<0.001	0.743
Finishing Phase									
Period 1 (day 0–23)									
Ending BW, kg	354	357	3.7	346	365	3.7	0.544	<0.001	0.618
ADG ^4^, kg	1.46	1.60	0.055	1.60	1.45	0.055	0.079	0.051	0.246
DMI ^5^, kg	6.47	6.02	0.271	6.94	5.56	0.271	0.243	<0.001	0.743
G:F ^6^	0.25	0.26	0.002	0.22	0.29	0.002	0.825	0.105	0.148
Period 2 (day 23–51)									
Ending BW, kg	402	403	4.5	385	421	4.5	0.915	<0.001	0.255
ADG ^4^, kg	1.72	1.65	0.055	1.37	2.00	0.055	0.312	<0.001	0.054
DMI ^5^, kg	7.40	7.22	0.328	7.35	7.26	0.328	0.706	0.843	0.960
G:F ^6^	0.21	0.21	0.004	0.17	0.27	0.004	0.566	<0.001	0.065
Period 3 (day 51–78)									
Ending BW, kg	448	451	5.0	428	471	5.0	0.651	<0.001	0.629
ADG ^4^, kg	1.68	1.77	0.054	1.60	1.84	0.054	0.224	0.002	0.071
DMI ^5^, kg	8.47	8.36	0.378	8.39	8.48	0.374	0.881	0.852	0.973
G:F ^6^	0.19	0.20	0.004	0.18	0.21	0.004	0.435	0.033	0.319
Period 4 (day 78–106)									
Ending BW, kg	502	507	5.27	486	524	5.27	0.499	<0.001	0.612
ADG ^4^, kg	1.96	2.02	0.057	2.07	1.91	0.057	0.416	0.047	0.874
DMI ^5^, kg	11.13	11.07	0.275	10.81	11.39	0.275	0.866	0.143	0.880
G:F ^6^	0.18	0.17	0.004	0.19	0.16	0.004	0.902	0.007	0.727
Period 5 ^7^									
Ending BW, kg	579	590	6.95	555	614	6.86	0.241	<0.001	0.660
ADG ^4^, kg	1.43	1.49	0.046	1.43	1.47	0.046	0.416	0.764	0.067
DMI ^5^, kg	14.02	14.00	0.190	14.04	13.99	0.190	0.964	0.862	0.253
G:F ^6^	0.10	0.10	0.003	0.10	0.10	0.003	0.263	0.505	0.307

^1^ Diets formulated based on NRC (2000) requirements for dams fed either a concentrate or forage diet during mid- and late-gestation. ^2^ Probability of difference among least square means. ^3^ Standard error of the mean. ^4^ ADG calculated from end of previous period to end of current period. ^5^ DMI: Dry matter intake. ^6^ G:F. gain to feed ratio. ^7^ Final BW, ADG, DMI, and G:F calculated based on when each animal was harvested at either d 131, d 145, or d 180. however, no differences (*p* > 0.05) in ADG were detected between treatment groups in subsequent periods.

**Table 4:** Least square means for ultrasound measurements of progeny from dams fed a prepartum dietary carbohydrate source consisting of concentrate-based (Conc) or forage-based (For) diet during mid- and late-gestation.

	Treatment ^1^			Sex			*p*-Value ^3^		
	Conc	For	SEM ^2^	Heifers	Steers	SEM ^2^	Trmt	Sex	T × S
Initial ultrasound during backgrounding phase									
Backfat, mm	3.94	3.82	0.124	4.06	3.70	0.124	0.503	0.046	0.502
Muscle Depth, mm	40.18	39.66	0.926	39.67	40.17	0.926	0.692	0.700	0.028
Intramuscular fat,%	5.07	4.98	0.1104	5.07	4.97	0.110	0.557	0.539	0.486
Ultrasound during finishing phase									
Backfat, mm	6.69	6.52	0.249	6.69	6.52	0.249	0.663	0.622	0.265
Muscle Depth, mm	50.76	50.93	0.877	50.64	51.05	0.877	0.890	0.743	0.926
Intramuscular fat,%	4.25	4.28	0.065	4.31	4.22	0.064	0.711	0.339	0.172
Change between ultrasound periods									
Backfat, mm	2.75	2.69	0.226	2.63	2.81	0.226	0.802	0.576	0.405
Muscle Depth, mm	10.58	11.31	1.276	10.97	10.91	1.276	0.684	0.974	0.127
Intramuscular fat,%	−0.82	−0.72	0.123	−0.76	−0.78	0.123	0.546	0.945	0.975

^1^ Diets formulated based on NRC (2000) requirements for dams fed either a concentrate or forage diet during mid- and late-gestation. ^2^ Standard error of the mean ^3^ Probability of difference among least square means.

**Table 5:** Least square means for carcass characteristics and meat quality of progeny from dams fed a prepartum dietary carbohydrate source consisting of concentrate-based (Conc) or forage-based (For) diet during mid- and late-gestation.

	Treatment ^1^			Sex			*p*-Value ^2^		
	Conc	For	SEM ^3^	Heifers	Steers	SEM ^3^	Trmt	Sex	T × S
Hot carcass weight, kg	349	351	4.4	335	366	4.4	0.710	<0.001	0.299
Ribeye area, cm ^2^	85.8	87.7	1.23	83.2	89.7	1.35	0.271	0.006	0.889
12th rib fat thickness, cm	1.22	1.14	0.041	1.27	1.09	0.046	0.060	0.002	0.304
USDA Yield grade	3.0	2.8	0.08	3.0	2.8	0.09	0.084	0.070	0.811
Marbling score ^4^	537	539	13.9	563	513	15.7	0.909	0.013	0.699
L* ^5^	42.05	41.83	0.277	41.99	41.90	0.314	0.534	0.838	0.826
a* ^5^	25.27	24.59	0.138	25.25	24.60	0.156	<0.001	0.002	0.921
b* ^5^	10.45	10.03	0.093	10.46	10.02	0.105	<0.001	0.001	0.660
USDA Quality Grade ^6^									
Prime, %	5.22	9.14	4.821	9.21	5.17	4.454	0.588	0.615	0.963
Upper 2/3 Choice, %	53.00	50.66	8.423	65.66	37.72	9.184	0.865	0.272	0.864
Low Choice, %	36.19	30.95	8.136	20.16	50.18	10.510	0.715	0.267	0.635
USDA Yield Grade ^6^									
Yield Grade 2, %	57.55	61.62	8.006	50.95	67.69	8.328	0.761	0.384	0.556
Yield Grade 3, %	40.50	36.50	7.849	46.59	30.96	8.184	0.761	0.399	0.794

^1^ Diets formulated based on NRC (2000) requirements for dams fed either a concentrate or forage diet during mid- and late-gestation. ^2^ Probability of difference among least square means ^3^ Standard error of the mean. ^4^ Marbling score: 200 = Traces ^0^, 300 = Slight ^0^, 400 = Small ^0^, 500 = Modest ^0^. ^5^ Recorded 3 d postmortem; L*: 0 = Black, 100 = White; a*: Negative values = green; Positive values = red; b*: Negative values = blue; Positive values = yellow. ^6^ Calculated proportions of USDA Quality and Yield Grade (data did not converge for a quality grade of USDA Select, or USDA Yield Grade less than a 2 or greater than a 3).

**Table 6:** Least square means for meat characteristics of progeny from dams fed a prepartum dietary carbohydrate source consisting of concentrate-based (Conc) or forage-based (For) diet during mid- and late-gestation.

	Treatment ^1^			Sex			*p*-Value ^2^		
	Conc	For	SEM ^3^	Heifers	Steers	SEM ^3^	Trmt	Sex	T × S
Crude Fat, %	6.31	6.24	0.339	7.17	5.39	0.384	0.865	<0.001	0.621
Moisture, %	71.48	71.50	0.264	70.69	72.29	0.299	0.945	<0.001	0.728
WBSF ^4^, kg	3.48	3.60	0.128	3.38	3.71	0.137	0.480	0.068	0.637
Tenderness ^5^	12.43	12.85	0.285	12.87	12.41	0.318	0.263	0.284	0.833
Juiciness ^5^	10.98	11.49	0.295	11.33	11.14	0.330	0.192	0.665	0.328
Flavor ^5^	9.83	9.64	0.228	9.84	9.64	0.255	0.531	0.555	0.232

^1^ Diets formulated based on NRC (2000) requirements for dams fed either a concentrate or forage diet during mid- and late-gestation. ^2^ Probability of difference among least square means. ^3^ Standard error of the mean. ^4^ Warner-Bratzler Shear Force. ^5^ Strip loin samples were evaluated for juiciness (1 = extremely dry; 18 = extremely juicy), tenderness (1 = extremely tough; 18 = extremely tender), and beef flavor (1= extremely bland; 18 = extremely intense).

**Table 7:** Least squares means for the fatty acid composition (mg/g raw wet tissue) of progeny from dams fed a prepartum dietary carbohydrate source consisting of concentrate-based (Conc) or forage-based (For) diet during mid- and late-gestation.

	Treatment ^1^			Sex			*p*-Value ^2^		
Fatty Acid	Conc	For	SEM ^3^	Heifer	Steer	SEM ^3^	Trmt	Sex	T × S
C10:0	0.03	0.03	0.003	0.03	0.02	0.003	0.710	0.013	0.290
C12:0	0.04	0.04	0.003	0.05	0.04	0.003	0.540	0.100	0.466
C14:0	2.15	2.06	0.154	2.34	1.87	0.172	0.663	0.042	0.348
C15:0	0.29	0.30	0.024	0.32	0.27	0.027	0.846	0.105	0.629
C16:0	19.37	19.43	1.410	20.58	18.23	1.572	0.974	0.264	0.477
C17:0	0.86	0.89	0.079	0.94	0.81	0.088	0.742	0.250	0.853
C18:0	10.33	10.73	0.788	10.45	10.61	0.879	0.697	0.896	0.495
C20:0	0.05	0.04	0.006	0.05	0.04	0.007	0.452	0.103	0.660
C14:1n5	0.57	0.50	0.042	0.62	0.46	0.047	0.204	0.017	0.402
C16:1n7	2.15	1.95	0.134	2.35	1.76	0.150	0.264	0.005	0.295
C16:1trans	0.24	0.25	0.014	0.25	0.24	0.016	0.723	0.698	0.566
C18:1n9	27.24	27.33	1.909	29.34	25.23	2.128	0.970	0.152	0.593
C18:1trans	2.58	2.41	0.203	2.47	2.52	0.226	0.517	0.853	0.467
C18:1n7	0.94	1.10	0.104	1.16	0.89	0.116	0.230	0.088	0.603
C18:2trans	0.004	0.003	0.0001	0.004	0.003	0.0006	0.628	0.596	0.245
C18:2n6	2.96	2.63	0.170	2.80	2.79	0.190	0.147	0.978	0.657
C18:3n6	0.02	0.02	0.001	0.01	0.02	0.001	0.766	0.201	0.806
C18:3n3	0.27	0.24	0.012	0.25	0.25	0.014	0.051	0.916	0.948
C20:2	0.06	0.05	0.004	0.06	0.05	0.005	0.638	0.240	0.921
C20:3n6	0.01	0.01	0.001	0.01	0.01	0.001	0.210	0.901	0.749
C20:4n6	0.55	0.46	0.025	0.493	0.524	0.028	0.009	0.405	0.547
C22:3	0.01	0.01	0.001	0.01	0.01	0.001	0.056	0.721	0.855
C24:1n9	0.02	0.01	0.002	0.01	0.01	0.002	0.011	0.530	0.224
C22:5n3	0.02	0.01	0.003	0.02	0.02	0.003	0.007	0.329	0.544
C22:6n3	0.03	0.03	0.003	0.03	0.03	0.003	0.514	0.811	0.888
SFA	33.12	33.52	2.410	34.77	31.87	2.688	0.897	0.419	0.477
MUFA	34.45	34.21	2.248	36.97	31.69	2.506	0.937	0.119	0.651
PUFA	3.93	3.47	0.192	3.69	3.71	0.214	0.068	0.958	0.767

^1^ Diets formulated based on NRC (2000) requirements for dams fed either a concentrate or forage diet during mid- and late-gestation. ^2^ Probability of difference among least square means. ^3^ Standard error of the mean.

**Table 8:** Least squares means for the fatty acid composition (%, g/100 g total fatty acids) of progeny from dams fed a prepartum dietary carbohydrate source consisting of concentrate-based (Conc) or forage-based (For) diet during mid- and late-gestation.

	Treatment ^1^			Sex			*p-*Value ^2^		
Fatty Acid	Conc	For	SEM ^3^	Heifer	Steer	SEM ^3^	Trmt	Sex	T × S
C10:0	0.04	0.04	0.004	0.05	0.04	0.004	0.863	0.130	0.303
C12:0	0.06	0.06	0.004	0.07	0.06	0.004	0.689	0.348	0.349
C14:0	2.97	2.90	0.082	3.08	2.79	0.092	0.508	0.021	0.202
C15:0	0.40	0.42	0.017	0.43	0.39	0.019	0.464	0.096	0.988
C16:0	26.84	27.18	0.380	27.18	26.83	0.424	0.491	0.540	0.403
C17:0	1.18	1.24	0.058	1.25	1.17	0.065	0.410	0.324	0.564
C18:0	14.38	14.80	0.360	13.76	15.41	0.401	0.373	0.003	0.886
C20:0	0.07	0.06	0.007	0.07	0.06	0.008	0.569	0.330	0.269
C14:1n5	0.81	0.73	0.041	0.82	0.71	0.045	0.158	0.082	0.389
C16:1n7	3.05	2.85	0.121	3.15	2.75	0.135	0.194	0.032	0.313
C16:1trans	0.34	0.34	0.010	0.33	0.35	0.011	0.670	0.083	0.867
C17:1	0.99	0.96	0.038	1.05	0.89	0.042	0.497	0.008	0.593
C18:1n9	38.03	38.32	0.616	38.76	37.59	0.0687	0.715	0.203	0.925
C18:1trans	3.62	3.41	0.170	3.27	3.76	0.190	0.343	0.057	0.094
C18:1n7	1.43	1.53	0.152	1.61	1.35	0.170	0.581	0.254	0.609
C18:2trans	0.005	0.005	0.0006	0.005	0.005	0.0007	0.814	0.847	0.213
C18:2n6	4.29	3.88	0.204	3.83	4.35	0.228	0.128	0.095	0.461
C18:3n6	0.02	0.02	0.002	0.02	0.03	0.002	0.346	0.078	0.348
C18:3n3	0.42	0.36	0.031	0.37	0.41	0.034	0.134	0.304	0.769
C20:2	0.09	0.08	0.007	0.08	0.10	0.008	0.428	0.936	0.720
C20:3n6	0.02	0.02	0.001	0.02	0.02	0.002	0.456	0.371	0.808
C20:4n6	0.83	0.70	0.053	0.70	0.83	0.059	0.057	0.120	0.912
C22:3	0.02	0.02	0.002	0.02	0.02	0.002	0.046	0.481	0.797
C24:1n9	0.02	0.01	0.003	0.02	0.02	0.003	0.003	0.639	0.323
C22:5n3	0.04	0.02	0.004	0.03	0.03	0.004	0.007	0.497	0.906
C22:6n3	0.05	0.05	0.006	0.04	0.05	0.007	0.384	0.229	0.936
SFA	45.94	46.70	0.681	45.89	46.75	0.681	0.390	0.397	0.516
MUFA	48.28	48.15	0.627	49.00	47.43	0.699	0.876	0.096	0.649
PUFA	5.78	5.15	0.275	5.11	5.82	0.307	0.080	0.086	0.568
PUFA:SFA	0.13	0.11	0.007	0.11	0.13	0.007	0.088	0.163	0.560
n6:n3	11.21	11.55	0.598	11.17	11.60	0.666	0.655	0.628	0.605
All Lipid	71.50	71.21	4.669	75.43	67.27	5.206	0.962	0.242	0.567

^1^ Diets formulated based on NRC (2000) requirements for dams fed either a concentrate or forage diet during mid- and late-gestation. ^2^ Probability of difference among least square means. ^3^ Standard error of the mean.

## Data Availability

The data presented in this study are available on request from the corresponding author.
